# Integrative taxonomy resolves taxonomic uncertainty for freshwater mussels being considered for protection under the U.S. Endangered Species Act

**DOI:** 10.1038/s41598-018-33806-z

**Published:** 2018-10-26

**Authors:** Nathan A. Johnson, Chase H. Smith, John M. Pfeiffer, Charles R. Randklev, James D. Williams, James D. Austin

**Affiliations:** 1 U.S. Geological Survey, Wetland and Aquatic Research Center, Gainesville, FL 32653 USA; 2 Baylor University, Biology Department, Waco, TX 76798 USA; 3 University of Florida, Florida Museum, Gainesville, FL 32611 USA; 4 Texas A&M Natural Resources Institute and AgriLife Research Center, Dallas, TX 75252 USA; 5 Wildlife Ecology and Conservation, University of Florida, Gainesville, FL 32601 USA

## Abstract

Objectively delimiting species boundaries remains an important challenge in systematics and becomes urgent when unresolved taxonomy complicates conservation and recovery efforts. We examined species boundaries in the imperiled freshwater mussel genus *Cyclonaias* (Bivalvia: Unionidae) using morphometrics, molecular phylogenetics, and multispecies coalescent models to help guide pending conservation assessments and legislative decisions. Congruence across multiple lines of evidence indicated that current taxonomy overestimates diversity in the *C. pustulosa* species complex. The only genetically and morphologically diagnosable species in the *C. pustulosa* species complex were *C. pustulosa* and *C. succissa* and we consider *C. aurea*, *C. houstonensis, C. mortoni*, and *C. refulgens* to be synonyms of *C. pustulosa*. In contrast, all three species in the *C. nodulata* complex (*C. necki*, *C. nodulata*, and *C. petrina*) were genetically, geographically, and morphologically diagnosable. Our findings have important conservation and management implications, as three nominal species (*C. aurea*, *C. houstonensis*, and *C. petrina*) are being considered for protection under the Endangered Species Act.

## Introduction

A robust taxonomy has profound implications for inferring common biological characteristics, understanding shared organismal responses, and is required to effectively set conservation priorities^1,2^. Methods used to delineate species continue to evolve, and arguments often reflect conflicting interpretations of available data types (e.g. morphological vs. molecular). Model-based approaches, such as multispecies coalescent models (MSC), have been increasingly utilized to delimit species boundaries^3–9^. Recent empirical studies, however, have criticized MSC models for identifying population structure rather than species boundaries^10–13^, which suggests caution is prudent when basing species hypotheses solely on MSC models. Recent authors have called for more integrative approaches that draw inference from multiple independent lines of evidence^14–21^. This has developed in part from the recognition that morphological characters or geographic distributions alone are not necessarily diagnostic at the generic and species levels^8,9,22–24^.

An example of a species-rich taxonomic group that has been characterized historically based on geographic patterns and phenotypic characters is freshwater mussels of the Unionidae, which is among the most critically endangered groups globally^25^. At least 10% of the unionid fauna in the United States are extinct, and 65% of the remaining species are considered imperiled^26,27^. Conservation efforts focused on many freshwater mussel taxa are complicated by taxonomic uncertainty that stems from high intraspecific variation and limited discrete morphological characteristics that would enable species diagnosis. Modern systematic research has been an important resource to the freshwater mussel conservation community by demonstrating that many traditional species-level hypotheses have both under- and overestimated species diversity, resulting in more accurate assessments of species conservation status^8,9,22,28–30^.

**Figure 1:**
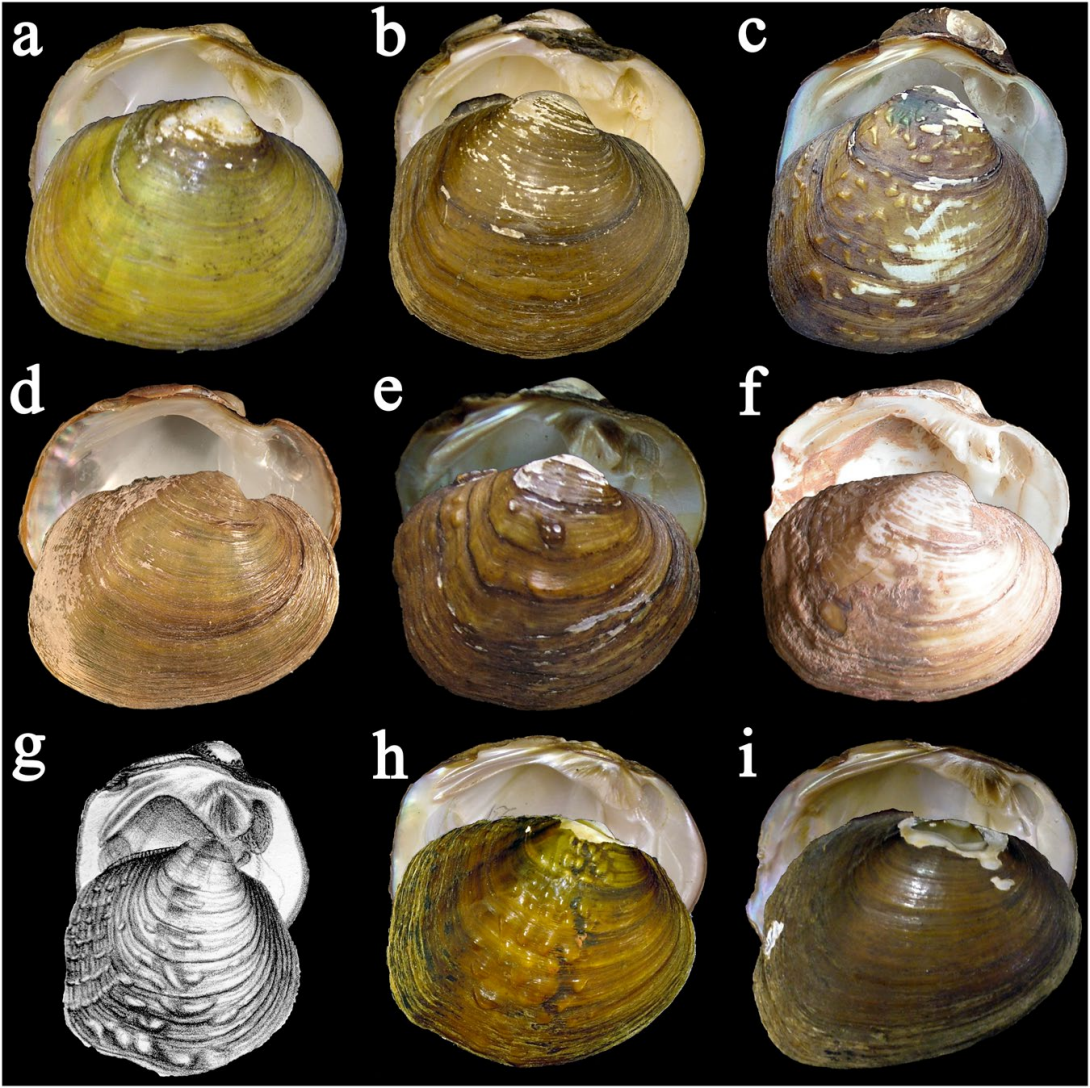
Photographs or illustrations of specimens representing members of the *Cyclonaias nodulata* and *Cyclonaias pustulosa* species complexes. (**a**) *Cyclonaias aurea* (Lea, 1859) holotype by monotypy USNM 84572, length 38 mm (**b**) *Cyclonaias houstonensis* (Lea, 1859) lectotype USNM 857680, length 33 mm (**c**) *Cyclonaias mortoni* (Conrad 1836) holotype by monotypy ANSP 10287, length 45 mm (**d**) *Cyclonaias necki* Burlakova *et al*. 2018, UF 439323, length 39 mm (**e**) *Cyclonaias nodulata* (Rafinesque, 1820) lectotype ANSP 20225, length 51 mm (**f**) *Cyclonaias petrina* (Gould, 1855) holotype by monotypy MCZ 169291, length 38 mm (**g**) *Cyclonaias pustulosa* (Lea, 1831) type not found, figured by Lea 1831: pl. 7, fig. 7, length 53 mm (**h**) *Cyclonaias refulgens* (Lea, 1868) lectotype USNM 84290, length 43 mm; and (**i**) *Cyclonaias succissa* (Lea, 1852) holotype by monotypy USNM 84574, length 43 mm. Photos a, b, c, e, f, and i courtesy of Kevin Cummings and Dan Graf (http://mussel-project.uwsp.edu/); photo d by Nathan Johnson.

North American unionids in the tribe Quadrulini Ihering, 1901 have been the focus of several generic, species, and population-level genetic studies^8,31–34^ but a comprehensive sampling using multiple, independently evolving molecular markers is lacking. The most recent nomenclatural review of freshwater mussels of the United States and Canada resolved generic confusion in the tribe Quadrulini and recognized 14 species in the genus *Cyclonaias* Pilsbry *in* Ortmann and Walker, 1922^35^. However, species boundaries in *Cyclonaias* remain largely untested and are complicated by a variety of morphological and geographic forms that have perplexed systematists for decades^36–40^ (Fig. 1).

Several nominal taxa of the genus *Cyclonaias* that occupy Gulf of Mexico drainages and lower sections of the Interior Basin of North America have been recognized as species distinct from *Cyclonaias pustulosa* (Lea, 1831). Phylogenetic studies placed *Cyclonaias aurea* (Lea, 1859), *Cyclonaias mortoni* (Conrad, 1836), *Cyclonaias refulgens* (Lea, 1868), *C. pustulosa*, and *Cyclonaias succissa* (Lea, 1852) together within the *C. pustulosa* species complex^32,41^, but the species boundaries within this group remain largely untested. Previous assessments reported the sympatric occurrence of several species in the *C. pustulosa* complex in western Gulf of Mexico drainages^36,37^. Most recently, the distributions of nominal species in the *C. pustulosa* complex in western Gulf of Mexico drainages have been revised with all species considered allopatric and previous reports of sympatry attributed to misidentifications^38^. One phylogenetic study^32^ also revealed the close relationship between *Cyclonaias nodulata* (Rafinesque, 1820) and *Cyclonaias petrina* (Gould, 1855) and advocated for denser intraspecific sampling before delineating species boundaries within this species complex, which we refer to as the *C. nodulata* species complex (Fig. 1). Of particular importance is the taxonomic validity of three species being considered for protection under the Endangered Species Act (ESA)^42^: *C. aurea*, *C. houstonensis*, and *C. petrina*.

In our study, we implement an integrative taxonomic approach that utilized multi-locus sequence data, morphometric analyses, and geographic distributions to investigate species boundaries in both the *C. nodulata* and *C. pustulosa* species complexes. Our findings support that four geographically isolated taxa (*C. aurea*, *C. houstonensis*, *C. mortoni*, and *C. refulgens*) are not valid species and are considered here as synonyms of *C. pustulosa*. Within the *C. nodulata* complex, we provide extensive molecular, morphological, and biogeographical evidence for *C. necki*, a recently described species^43^. In order to clarify the distribution, phylogenetic position, and morphological variation, we redescribe *C. necki* based on our more robust integrative dataset. Our findings have important conservation and management implications and are likely to impact ESA listing decisions for at least three nominal species (*C. aurea*,*C. houstonensis*, and *C. petrina* s.s.).

## Results

### Taxon Sampling and Molecular Analyses

**Figure 2:**
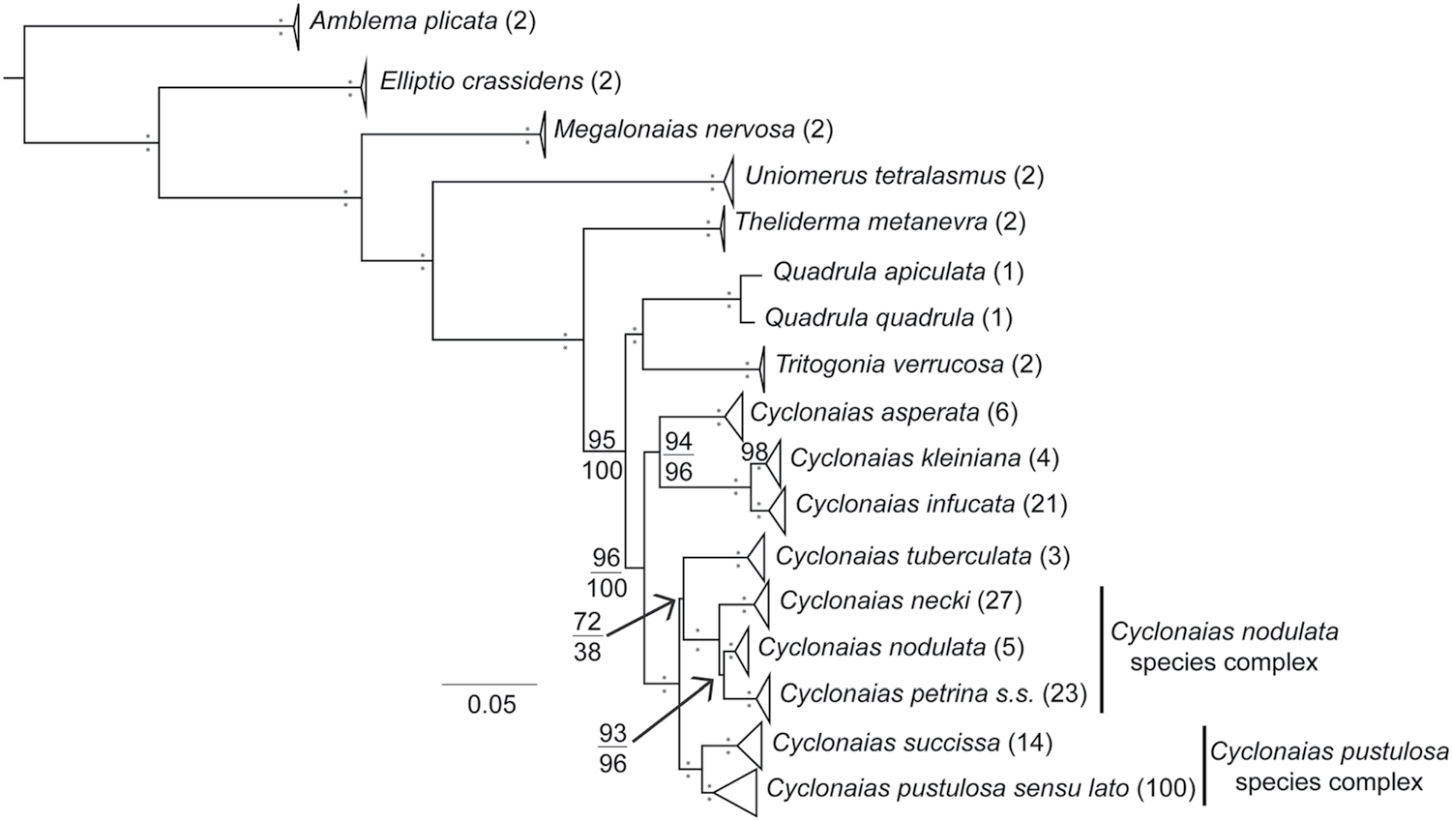
Maximum likelihood (ML) phylogeny based on concatenated mtDNA and nDNA datasets for Quadrulini. Nodes are collapsed into species-level clades. Asterisks above and below branches represent ≥99% ultrafast bootstrap and 0.99 posterior probability support, respectively. Number in parentheses after taxon name indicates sample size. The fully resolved phylogeny with ultrafast bootstraps and posterior probability support values are available as Supplementary Figs S1 and S2, respectively.

We generated and analyzed 391 CO1, 391 ND1, and 217 ITS1 DNA sequences for this investigation. Collection details, museum catalog numbers, and GenBank accession numbers are presented in Supplementary Table S1 (also available at https://doi.org/10.5066/P9SRSHV2). Our three-locus molecular matrix consisted of 217 individuals representing 8 genera and 21 recognized species (Table 1). Each taxon was represented by CO1 (avg. ≈ 642 nucleotides [nt]), ND1 (avg. ≈ 797 nt), and ITS1 (951 nt with avg. ≈ 49.13% gaps) and the concatenated three-locus alignment consisted of 2397 nt. Protein coding mtDNA genes did not contain any gaps or stop codons. The large proportion of gaps in the ITS1 alignment was a consequence of partial duplication in the gene region (294–298 nt) found in *Cyclonaias tuberculata*, which was previously reported^33^. Five partitions and nucleotide substitution models were selected by Partitionfinder for implementation in both IQ-TREE and BEAST: CO1 and ND1 1st position- TrNef+I+G, CO1 and ND1 2nd position- HKY+I+G, CO1 3rd position- HKY+G, ND1 3rd position- TrN+G, and ITS1- K80+I+G. To reduce redundancy of IQ-TREE and BEAST analyses, invariant sites were not modeled in instances when a gamma distribution for rate heterogeneity was estimated^44,45^. Convergence of BEAST runs was supported by ESS > 200 for all parameters. We present the ML phylogenetic reconstruction of the concatenated 3-gene matrix containing ML and BI nodal support values (Fig. 2; Supplementary Figs S1 and S2; also available at https://doi.org/10.5066/P9SRSHV2).

**Table 1:** Taxa sampled, drainage of collection, and number of sequences for all individuals included in molecular analyses.

Taxa	Drainage	CO1 & ND1	ITS1
**Tribe Amblemini**			
*Amblema plicata*	Colorado	2	2
**Tribe Pleurobemini**			
*Elliptio crassidens*	Ohio	1	1
	Pearl	1	1
**Tribe Quadrulini**			
Cyclonaias aurea**	Guadalupe	30	9
	Nueces	39	7
*Cyclonaias asperata*	Mobile	6	6
Cyclonaias necki*	Guadalupe	33	27
Cyclonaias houstonensis**	Brazos	18	12
	Colorado	14	7
*Cyclonaias infucata*	Apalachicola	16	16
	Ochlockonee	5	5
*Cyclonaias kleiniana*	Suwannee	4	4
Cyclonaias mortoni**	Neches	26	10
	Sabine	8	6
	San Jacinto	9	0
	Trinity	15	9
Cyclonaias nodulata*	Neches	3	0
	Ouachita	5	4
	Red	1	0
	Salt	5	1
*Cyclonaias petrina* s.s.*	Colorado	33	23
*Cyclonaias pustulosa* s.l.**	Neosho	4	2
	Ohio	9	5
	Osage	4	2
	Ouachita	16	8
	Red	26	11
	St. Croix	5	3
	St. Francis	11	4
Cyclonaias refulgens**	Pascagoula	5	3
	Pearl	5	2
Cyclonaias succissa**	Choctawhatchee	19	9
	Escambia	3	2
	Yellow	3	3
*Cyclonaias tuberculata*	Tennessee	3	3
*Megalonaias nervosa*	Guadalupe	1	1
	Ohio	1	1
*Quadrula apiculata*	Rio Grande	1	1
*Quadrula quadrula*	Ohio	1	1
*Theliderma metanevra*	Ohio	1	1
	Tennessee	1	1
*Tritigonia verrucosa*	Ohio	1	1
	Red	1	1
*Uniomerus tetralasmus*	Bayou Pierre	1	1
	Colorado	1	1
Total		397	217

*denotes taxa within the *Cyclonaias nodulata* complex and **denotes taxa within the *Cyclonaias pustulosa* complex.

**Figure 3:**
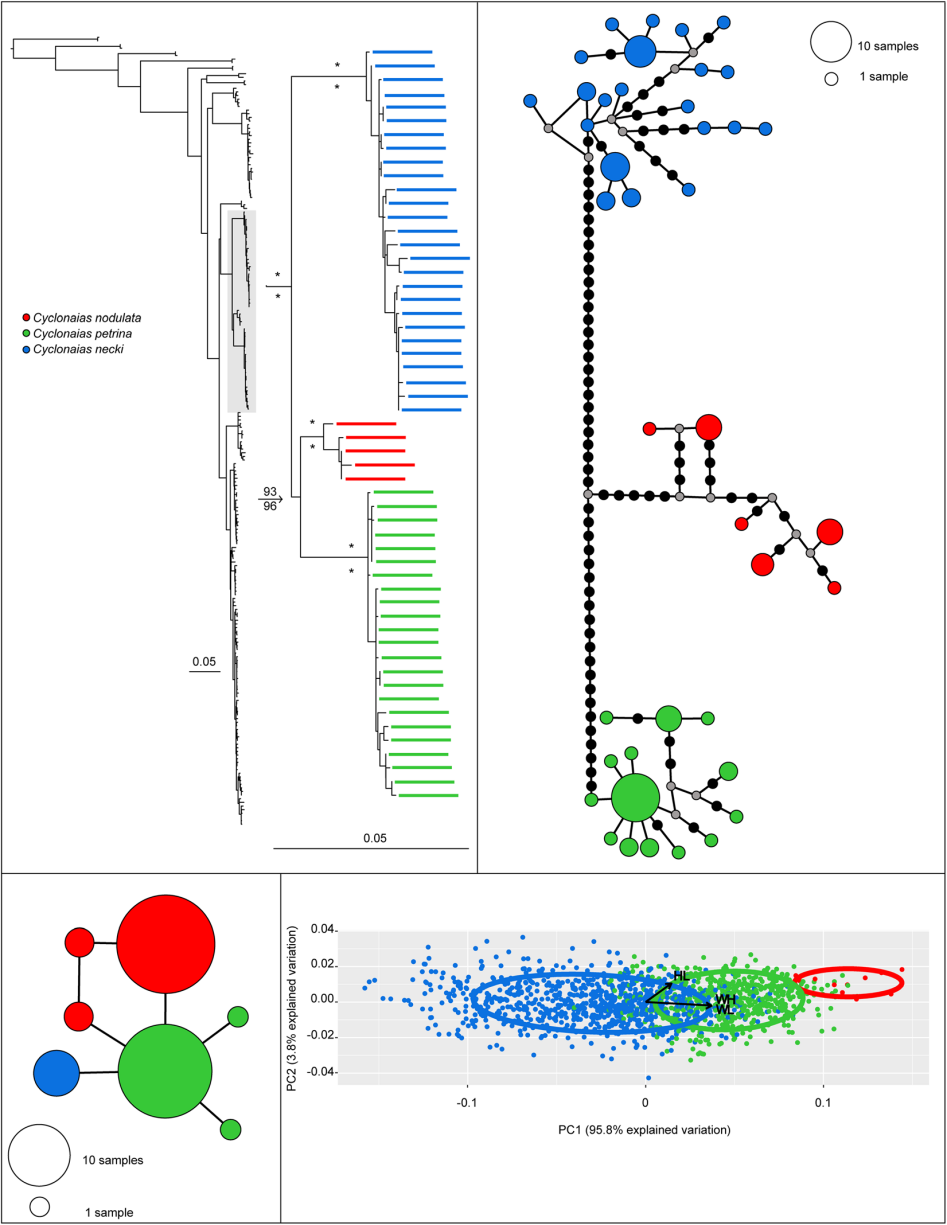
Comparison of results for members of the *Cyclonaias nodulata* species complex. Clockwise from the top-left panel: Most likely topology and expanded phylogeny based on CO1, ND1, and ITS1 sequences; CO1 + ND1 haplotype network; PCA plots with 95% CI ellipses and arrows for biplot variables (HL, height/length; WL, width/length; WH, width/height); and ITS1 haplotype network. Colors indicate the following taxa: red (*Cyclonaias nodulata*); green (*Cyclonaias petrina*); blue (*Cyclonaias necki*.). On the haplotype networks, black dots represent mutations and gray dots represent unsampled haplotypes. Asterisks above and below branches on the phylogeny represent ≥99% ultrafast bootstrap and 0.99 posterior probability support, respectively. The fully resolved phylogeny with ultrafast bootstraps and posterior probability support values are available as Supplementary Figs S1 and S2, respectively.

**Figure 4:**
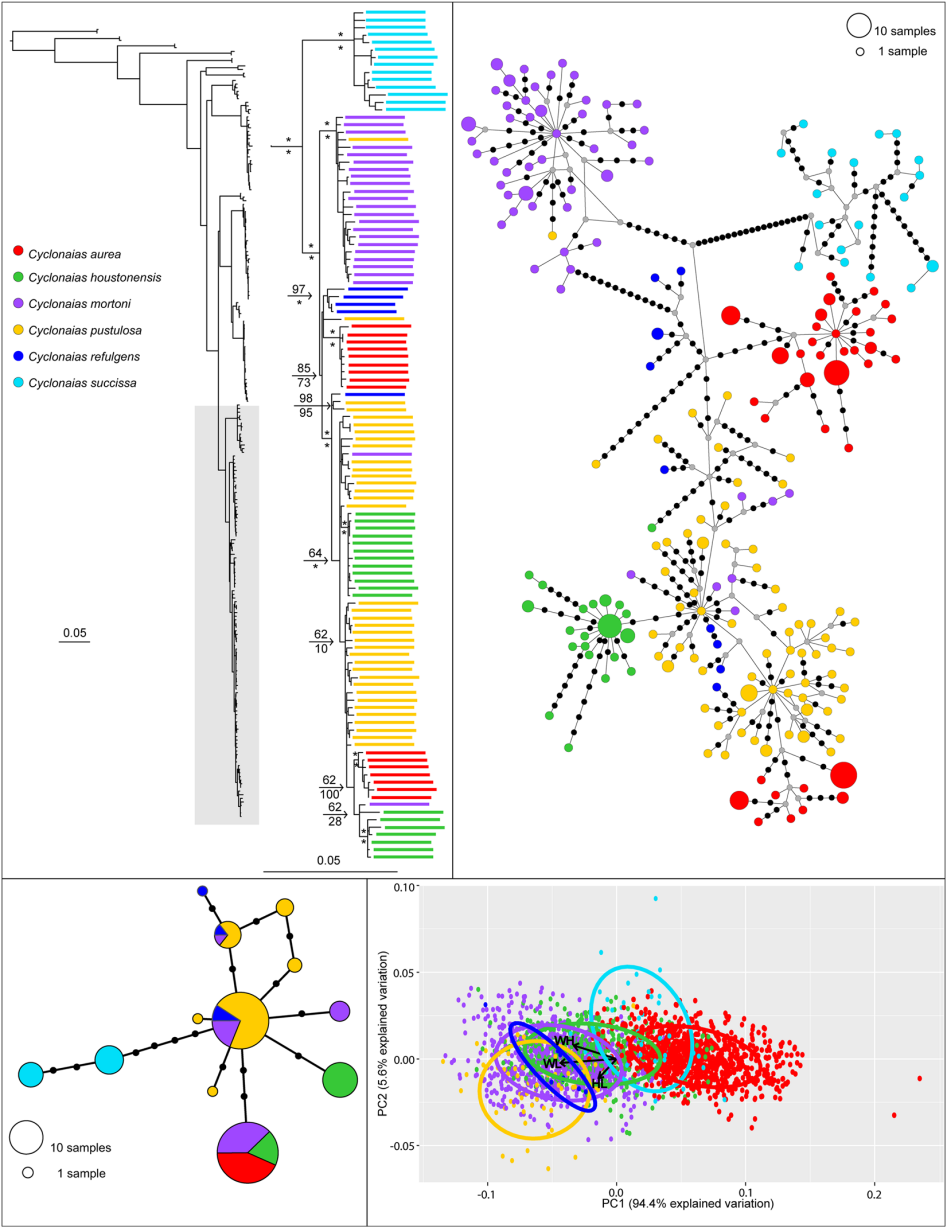
Comparison of results for members of the *Cyclonaias pustulosa* species complex. Clockwise from top-left panel: Most likely topology and expanded phylogeny based on CO1, ND1, and ITS1 sequences; CO1 + ND1 haplotype network; PCA plots with 95% CI ellipses and arrows for biplot variables (HL, height/length; WL, width/length; WH, width/height); and ITS1 haplotype network. Colors indicate the following taxa: red (*Cyclonaias aurea*); green (*Cyclonaias houstonensis*); purple (*Cyclonaias mortoni*); yellow (*Cyclonaias pustulosa*); blue (*Cyclonaias refulgens*); cyan (*Cyclonaias succissa*). On the haplotype networks, black dots represent mutations and gray dots represent unsampled haplotypes. Asterisks above and below branches on the phylogeny represent ≥99% ultrafast bootstrap and 0.99 posterior probability support, respectively. The fully resolved phylogeny with ultrafast bootstraps and posterior probability support values are available as Supplementary Figs S1 and S2, respectively.

Phylogenetic analyses recovered *C. nodulata* nested between two monophyletic and geographically isolated clades representing *C. petrina* s.s. and *C. necki* (Fig. 3). In contrast, five of the six recognized species in the *C. pustulosa* species complex were not monophyletic in the optimal topology (Fig. 4). Specifically, *C. succissa* was sister to a clade containing *C. aurea, C. houstonensis*, *C. mortoni*, *C. pustulosa*, and *C. refulgens*. For the *C. nodulata* complex, totals of 80 and 55 individuals were included in the mtDNA and ITS1 haplotype networks, respectively (Fig. 3). Three geographically isolated groups were recovered in both networks: *C. petrina* from the Colorado River basin, *C. necki* from the Guadalupe River basin, and *C. nodulata* from the Neches, Ouachita, Red, and Salt river basins. For the *C. pustulosa* species complex, 263 and 114 individuals were included in the mtDNA and ITS1 haplotype networks, respectively (Fig. 4). *Cyclonaias succissa* was molecularly diagnosable from other taxa and clearly divergent in both the mtDNA and ITS1 haplotype networks. All other species shared ITS1 haplotypes and showed weak phylogeographic structuring among mtDNA haplotypes.

**Figure 5:**
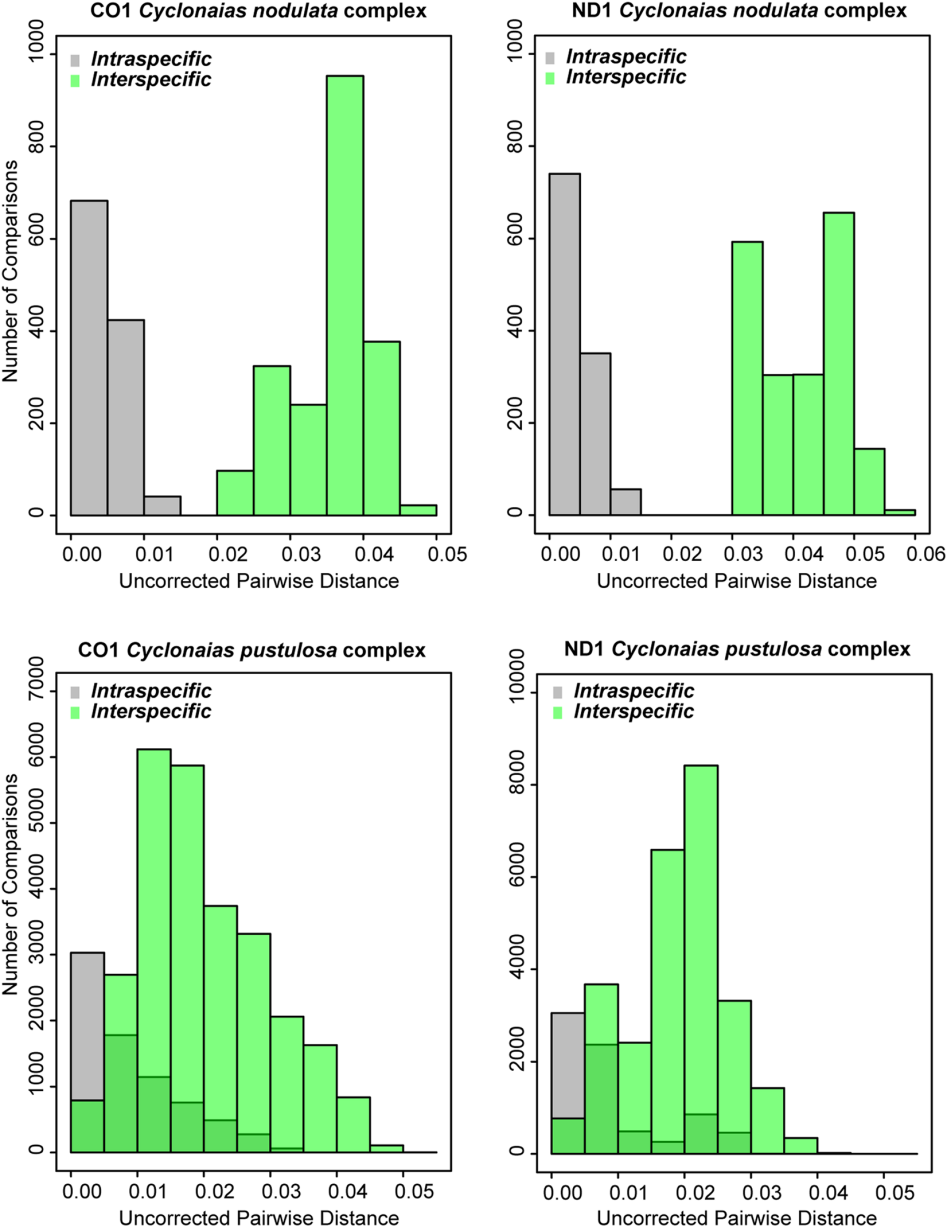
Histograms illustrating the distribution of all intraspecific and interspecific pairwise uncorrected-p distances for *Cyclonaias nodulata* complex (top) and *Cyclonaias pustulosa* complex (bottom) based on CO1 (left) and ND1 (right).

We observed no overlap between intraspecific variation and interspecific divergence in genetic distance among members of the *C. nodulata* complex (Fig. 5). Additionally, all three clades contained diagnostic nucleotides: *C. petrina* (CO1/ND1/ITS1 = 4/16/2), *C. necki* (CO1/ND1/ITS1 = 10/12/3), and *C. nodulata* (CO1/ND1/ITS1 = 6/5/3). However, uncorrected p-distances show a high degree of overlap between intraspecific variation and interspecific divergence among members of the *C. pustulosa* complex, with the exception of *C. succissa* (Fig. 5), which also exhibited diagnostic nucleotides (CO1/ND1/ITS1 = 3/4/6). None of the other taxa were molecularly diagnosable. The AMOVA results parallel the levels of genetic distances observed in each species complex (Table 2). The AMOVA for members of the *C. pustulosa* complex indicated that genetic variation within species was roughly equal to variation between species, with 52.42% and 51.32% of the variation within species, and 47.58% and 48.68% between all species species for CO1 and ND1, respectively. In contrast, AMOVA between members of the *C. nodulata* complex revealed high levels of genetic structuring, with 87.45% and 88.98% of the variation between the three species groups and 12.55% and 11.02% within species groups for CO1 and ND1, respectively.

**Table 2:** Analysis of molecular variance (AMOVA) among members of the *Cyclonaias nodulata* and *Cyclonaias pustulosa* species complexes. Samples were grouped according to current taxonomy.

Source of variation	Percentage of variance	
	CO1	ND1
*Cyclonaias nodulata* complex		
Among groups	87.45	88.98
Within groups	12.55	11.02
*Cyclonaias pustulosa* complex		
Among groups	47.58	48.68
Within groups	52.42	51.32

All values were significant (P < 0.0001).

### Morphometric Analyses

We measured a total of 3800 individuals from museum and field collections, representing members of the *C. nodulata* (n = 1387) and *C. pustulosa* (n = 2413) complexes: *C. necki* (n = 849), *C. petrina* (n = 527), *C. nodulata* (n = 11), *C. aurea* (n = 868), *C. houstonensis* (n = 604), *C. mortoni* (n = 796), *C. pustulosa* (n = 95), *C. refulgens* (n = 10), and *C. succissa* (n = 40). Measurement data and details on collection location for all specimens used in the morphometric analyses are available as Supplemental Table S2 (also available at https://doi.org/10.5066/P9SRSHV2). PCA eigenvalues explained 99.6% and 100% of the total variability between members of the *C. nodulata* and *C. pustulosa* complexes, respectively (Figs 3 and 4). The PCA for the *C. nodulata* complex revealed high levels of morphological variation among individuals within three relatively distinct groups; *C. necki*, *C. petrina*, and *C. nodulata*. Cross-validated DA scores provided an overall classification accuracy of 80.1% (*C. petrina* = 77.8%; *C. necki* = 81.3%; *C. nodulata* = 100%). PCA for the *C. pustulosa* complex showed high levels of morphological overlap between currently recognized species. Cross-validated DA scores provided an overall classification accuracy of 50.48% (*C. aurea = *74.3%; *C*. *houstonensis* = 47.2%; *C. mortoni* = 25.9%; *C. pustulosa* = 61.1%; *C. refulgens* = 40.0%; and *C. succissa* = 50.0%).

### Multispecies Coalescent Delimitation

The molecular matrix for members of the *C. nodulata* complex consisted of 58 individuals aligned to 2307 nt and the molecular matrix for the *C. pustulosa* complex contained 114 individuals aligned to 2018 nt. Five partitions and substitution models were selected for STACEY by Partitionfinder and differed slightly between the two matrices (i.e., *C. nodulata* complex: CO1 and ND1 1st position- K80+I, CO1 and ND1 2nd position- HKY, CO1 3rd position- HKY+G, ND1 3rd position- HKY+G, and ITS1- K80+I; *C. pustulosa* complex: CO1 and ND1 1st position- TrN+I, CO1 and ND1 2nd position- HKY+I, CO1 3rd position- HKY+G, ND1 3rd position- HKY+G, and ITS1- K80+I+G). In the STACEY analyses for the *C. pustulosa* species complex, invariant sites were not modeled in the ITS1 partition; instead, the gamma distribution for rate heterogeneity was estimated^45^. All STACEY analyses were effectively sampled with all ESS values > 200. STACEY supported 4 species clusters in the *C. nodulata* complex. The four clusters depicted were *C. necki*., *C. tuberculata*, *C. nodulata*, and *C. petrina*. For the *C. pustulosa* complex, STACEY could not reach a definitive consensus regarding the number of species clusters (probability for all species clusters < 0.00004%).

**Taxonomic Accounts**

**Family:** Unionidae Rafinesque, 1820

**Subfamily:** Ambleminae Rafinesque, 1820

**Tribe:** Quadrulini von Ihering, 1901

**Genus:** *Cyclonaias* Pilsbry *in* Ortmann and Walker, 1922

**Comments:** The genus *Cyclonaias* as presented by Williams *et al*.^35^ included 14 species. Based on our findings, we place four currently recognized species, *C. aurea*, *C. houstonensis*, *C. mortoni*, and *C. refulgens*, into the synonymy of *C. pustulosa* (Figs 4, 5, and Table 1 – full synonymy for *C*. *pustulosa* listed below).

**Type species:** *Obliquaria (Rotundaria) tuberculata* Rafinesque, 1820

**Redescription:** *Cyclonaias necki* Burlakova *et al*. 2018

**Common name:** Guadalupe Orb

**Type Material**

**Holotype:** NCSM 65378, length 42 mm, Texas, Victoria County, San Marcos River, between US90 and SR80, southwest of Luling, Caldwell/Guadalupe counties, Texas (N29.67078; W97.69561), 12 July 2011.

**Material Examined for Redescription:** For the *C. nodulata* complex, totals of 80 and 55 individuals were sequenced for mtDNA and ITS1 haplotype networks, respectively (Fig. 3). Additionally, morphological measurements were taken and analyzed for *C. necki* (n = 849), *C. petrina* (n = 527) and *C. nodulata* (n = 11). Complete details on all specimens are provided in Supplementary Table S1.

**Revised Diagnosis:** Specimens of *C. necki* are distinguished from *C. petrina* by having a shell that is more elongate and more compressed with less fluting along the posterior slope and a periostracum that is typically more yellow with subdued broken green rays (Fig. 1b). It also lacks the two rows of nodules present on the shell disk of *C. nodulata*. *Cyclonaias necki* also has 10 diagnostic nucleotides at CO1 (148:C, 273:T, 282:A, 291:G, 294:T, 328:C, 378:C, 468:A, 582:G, 666:A), 12 at ND1 (138:C, 233:C, 261:G, 303:G, 309:G, 327:T, 405:A, 435:G/A, 444:G/C, 509:A, 636:T, 739:C), and 3 diagnosable sites at ITS1 (96:-, 97:-, 494:A/G), which differentiate *C. necki* from its sister species, *C. petrina* and *C. nodulata*.

**Redescription:** Maximum shell length to 69 mm, height to 50 mm, and width to 30 mm. Shell subquadrate to subovate or nearly ovate in outline, often with slight corrugations or parallel ridges on the posterior slope that are sometimes obscured by the accumulation of precipitates on the posterior portion of the shell. Posterior ridge rounded, shells thick, solid, and moderately compressed laterally. Periostracum often with a cloth-like texture, yellow to tan or brown to black in color, sometimes with broken green rays or blotches on the disc or along the posterior slope. Nacre color white, usually iridescent posteriorly. Umbo high and usually extends well above hinge line. Umbo sculpture with 2–4 rows of nodules following a 45° angle relative to hinge line with cross-hatching in younger specimens. Left valve with two thick lateral teeth, straight to slightly curved; two low, robust pseudocardinal teeth. Right valve with single, lateral tooth, two pseudocardinal teeth; anterior tooth small relative to posterior tooth, both triangular. Umbo cavity deep, compressed, and extending well under the interdentum.

**Figure 6:**
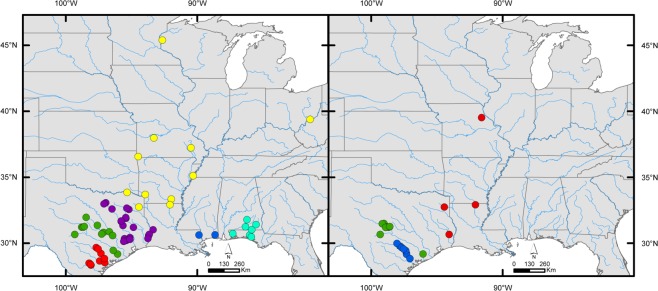
Map showing sampled localities for specimens used to generate molecular data for members of the *Cyclonaias pustulosa* species complex (left) and *Cyclonaias nodulata* species complex (right). Colors correspond to nominal taxa within each complex: *C. pustulosa* complex - red (*C. aurea*), green (*C. houstonensis*), purple (*C. mortoni*), yellow (*C. pustulosa*), blue (*C. refulgens*), and cyan (*C. succissa*); *C. nodulata* complex - red (*C. nodulata*), green (*C. petrina*), and blue (*C. necki*).

**Distribution:** *Cyclonaias necki* is endemic to the Guadalupe River drainage in Central Texas (Fig. 6). The historical distribution of *C. necki* in the Guadalupe River drainage is known from observations in the Guadalupe and Blanco rivers. In the Guadalupe River, *C. necki* was collected from Comal/Guadalupe (A. L. Fitzpatrick, BU-MMC_MO 33308 -A-B), Kendall (J. K. Strecker, BU-MMC_MO 33667 -A-B), Kerr^46^, and Victoria counties^46^. In the Blanco River, a major tributary of the San Marcos River, *C. necki* has been observed at several localities^47^, including a single specimen collected in Hays County (W.J. Williams, BU-MMC_MO 34296 -A-B).

In the San Antonio River drainage, historical records for *C. necki* are limited to a single specimen (UMMZ 77200) purportedly collected from Salado Creek, a tributary of the San Antonio River. Based on shell morphology, this specimen looks more like *C. petrina* from the Colorado River than *C. necki* from the Guadalupe, suggesting the locality information associated with this single specimen may be inaccurate. Furthermore, Strecker^46^ did not report *C. necki* from the San Antonio River basin. In the 1970s, Joseph Bergmann reported shell material, of unknown condition, of *C. necki* from the Medina River near Von Ormy and Macdona, TX, and Salado Creek near Fort Sam Houston^48^, but these collections have since been lost and so the identifications cannot be verified. Around the same time P. Barker, an amateur naturalist, reported collecting a single specimen of *C. necki* from Medina Reservoir^48^. This species is not known to occur in lakes or reservoirs so the true collection locality for this specimen remains in question. More recently, investigators reported finding shell fragments thought to be *C. necki* from the San Antonio River at the San Antonio River Walk^49^, but the weathered condition of these fragments precludes confident identification to any species.

Recent fieldwork in central Texas has led to the discovery of live individuals or very recently dead specimens of *C. necki* in the following rivers within the Guadalupe River drainage: San Marcos River in Caldwell, Guadalupe, Gonzales, and Hays counties; and Guadalupe River in Comal, Gonzales, Kerr, Kendall, and Victoria counties^48,50–52^ (Supplementary Table S1).

*Cyclonaias petrina* (Gould, 1855)

**Synonymy:**

*Unio petrinus* Gould, 1855: 228

*Unio bollii* Call, 1881: 390

**Common name:** Texas Pimpleback

**Type Material**

**Holotype:** MCZ 169291 (Fig. 1f), length 38 mm, Texas, Llanos River, collected by T. H. Webb.

**Material Examined:** All material examined available as Table S1.

**Diagnosis:** *Cyclonaias petrina* is distinguished from *C. necki* by having a shell that is thicker, less elongate, and less compressed with more fluting along the posterior slope and a periostracum that is typically more tan to brown, occasionally with vague green rays or blotches. Unlike *C. nodulata*, it lacks the two radiating rows of nodules located on the shell disk (Fig. 1). *Cyclonaias petrina* can be distinguished based on 4 diagnostic nucleotides at CO1 (132:A, 183:G, 186:G, 651:C), 16 at ND1 (27:C, 102:T, 241:A, 244:T, 256:T, 492:T, 537:C, 567:G, 573:A, 600:T, 648:T, 684:A, 720:T, 735:C, 753:T, 765:A), and 2 at ITS1 (96:A, 97:A), which differentiate *C. petrina* from both *C. necki* and *C. nodulata*.

**Material Examined:** Maximum shell length to 86 mm, height to 70 mm, and width to 46 mm. Shell subquadrate to subovate or nearly ovate in outline, often with slight corrugations or parallel ridges on the posterior slope that are sometimes obscured by the accumulation of precipitates on the posterior portion of the shell. Posterior ridge rounded, shells thick to moderately thin, and moderately compressed laterally. Periostracum yellow to tan or brown to black in color, sometimes with broken green rays or blotches on the disk or along the posterior slope. Nacre color white, usually iridescent posteriorly. Umbo high and usually extends well above the hinge line. Umbo sculpture with 2 to 4 rows of nodules following a 45° angle relative to the hinge line with cross-hatching in younger specimens. Left valve with two thick lateral teeth, straight to slightly curved; two low, robust pseudocardinal teeth. Right valve with single, lateral tooth, two pseudocardinal teeth; anterior tooth small relative to posterior tooth, both triangular. Umbo cavity deep, compressed, and extending well under the interdentum.

**Material Examined:** *Cyclonaias petrina* is endemic to the Colorado River drainage in central Texas, including the Llano, San Saba, and Pedernales rivers (Fig. 6). Historically, *C. petrina* was known from observations made in the mainstem of the Colorado River and several tributaries in the upper portions of the basin. Singley^53^ collected specimens of *C. petrina* in the Colorado River near Austin, and through secondhand observations, he reported several records from the Brazos and Trinity rivers. However, Singley^53^ and Frierson^54^ noted that investigators often confused *C. petrina* with other closely related species (e.g., *C. houstonensis*), particularly specimens with heavily eroded shells. Thus, specimens collected in the Brazos and Trinity rivers attributed to *Q. petrina* were misidentified *C. houstonensis* and *C. mortoni*, respectively. Others have reported *C. petrina* from the Colorado River near Austin (J.K. Strecker, BU-MMC_MO 33291 -A-B) and from a number of major tributaries in the basin: Llano River in Llano (W.T. Little, BU-MMC_MO 32982 -A-B; J. Dobie, AUM_2944, AUM_2975, AUM_4022, AUM_4046), Mason (A.L. Fitzpatrick, BU-MMC_MO 33549 -A-B), and Kimble counties (J. Dobie, AUM_4076); San Saba River in Menard (Strecker 1931, Cheathum *et al*. 1972, FWMSH_94V 2704) and McCulloch counties (A.L. Fitzpatrick, Strecker^46^); South Concho River in Tom Green County (Williams, Strecker 1931); Onion Creek in Travis County (J.D. Mitchell, Strecker^46^, and Pedernales River in Blanco County^48^.

Live individuals or very recently dead specimens of *C. petrina* have been reported recently from the following rivers in the Colorado River drainage: Elm Creek in Runnels County; Concho River in Concho County; Llano River in Mason County; San Saba River in Menard and San Saba counties; Colorado River in Colorado, San Saba, Mills, and Wharton counties^50,51,55^ (Supplementary Table S1). This indicates that *C. petrina* continues to persist within the Llano and San Saba rivers. For the Pedernales River, no live or shell material of *C. petrina* was observed despite it being collected from this river in the early 1970s.

*Cyclonaias pustulosa* (Lea, 1831)

**Synonymy:**

*Obliquaria* (*Quadrula*) *bullata* Rafinesque, 1820: 307–308

*Obliquaria (Quadrula) retusa* Rafinesque, 1820: 306, pl. 81, figs. 19–20

*Unio verrucosa* Valenciennes, 1827: 231, pl. 53, Fig. 2

*Unio premorsus* Rafinesque, 1831: 4

*Unio pustulosus* Lea, 1831: 76, pl. 7, fig. 7

*Unio nodulosus* Say, 1834: no pagination

*Unio prasinus* Conrad, 1834: 44, pl. 3, Fig. 1

*Unio schoolcraftensis* Lea, 1834: 37, pl. 3, fig. 9

*Unio mortoni* Conrad, 1836: 11, pl. 6, Fig. 1

*Unio dorfeuillianus* Lea, 1838: 73, pl. 17, fig. 54

*Unio turgidus* Lea, 1838: 11, pl. 5, fig. 11

*Unio nodiferus* Conrad, 1841: 19

*Unio pernodosus* Lea, 1845: 71, pl. 3, fig. 8

*Unio aureus* Lea, 1859: 112

*Unio houstonensis* Lea, 1859: 155

*Unio refulgens* Lea, 1868: 145

*Unio sphaericus* Lea, 1868: 319, pl. 51, fig. 132

*Unio petrinus* Frierson, 1927: 49

**Common name:** Pimpleback

**Type Material**

**Holotype:** Original figured type specimen not found, which was designated as a lectotype by Johnson (1974). The length of figured shell in original description reported as about 53 mm (Fig. 1g). Syntypes, ANSP 43058, are from Ohio.

Material Examined: All material examined available as Table S1.

**Diagnosis:** *Cyclonaias pustulosa* is difficult to distinguish from other species of the genus due to high variability in shell morphology and nacre color (Fig. 1). *Cyclonaias pustulosa* can be distinguished from *C. succissa* based on three diagnostic nucleotides at CO1 (28:T, 78:T, 529:T), four at ND1 (22:G, 111:T, 313:T, 657:T), and six at ITS1 (69:G, 71:C, 73:A, 81:C, 382:C, 502:T).

**Redescription:** Maximum shell length to 83 mm, height to 73 mm, and width to 52 mm. Shell subquadrate to subovate but usually ovate in outline, posterior margin rounded to truncated, posterior slope broad and flat with or without pustules or corrugations. Posterior ridge rounded, shells thick, solid, and moderately to greatly inflated. Shell disk smooth to heavily pustulated. Periostracum smooth to cloth-like, yellow to tan or brown to black in color, occasionally with a broad, broken green ray or blotches extending from the umbo to the posterior ventral margin. May also have green blotches on the posterior slope. Nacre color typically white, although can be light to dark purple. Umbo high, inflated, oriented anteriorly, and usually extends well above hinge line. Umbo sculpture with two to four course ridges, somewhat more nodulous along posterior ridge. Left valve with two thick, straight to slightly curved lateral teeth; two large, robust, erect, triangular pseudocardinal teeth. Right valve with single, lateral tooth, two pseudocardinal teeth, also triangular. The interdentum is moderately wide. Umbo cavity deep, somewhat compressed, and extending well under the interdentum.

**Distribution:** *Cyclonaias pustulosa* is known from the eastern reaches of the Great Lakes, Lake St. Clair and Lake Erie, and widespread throughout the Mississippi Basin from southern Minnesota south to Louisiana, and from western New York west to South Dakota^39,56^. Based on our findings, we have expanded the range of *C. pustulosa* to include Gulf Coast rivers from the Pascagoula Basin in Mississippi west to the Nueces Basin in southwest Texas (Fig. 6).

## Discussion

Our primary goal was to implement an integrative taxonomic approach^14,18,57^ that utilized multi-locus sequence data, morphometric analyses, and geographic distributions to investigate species boundaries in the genus *Cyclonaias*. For our assessment, we allowed current taxonomy^35,37,38,40,58^ to represent our null hypotheses regarding species-level boundaries. Based on this approach, we identified nine well-supported species-level clades, including two species complexes containing taxa of immediate conservation concern (Figs 3–5).

For the *C. nodulata* complex, both BI and ML analyses resolved *C. nodulata* nested between two monophyletic and geographically isolated clades representing *C. petrina* s.s. and *C. necki* (Figs. 2 and 3). A clear gap between intraspecific variation and interspecific divergence among the three geographically isolated clades (Fig. 5) was exhibited by mtDNA sequences, indicative of species-level divergence and similar to values reported for several other freshwater mussel species^8,9,22,24,32,59–61^. Sequence divergence at ITS1 was lower relative to both mtDNA genes but consistent with patterns observed in previous studies utilizing these loci^8,9,24^. Morphometric analyses revealed little overlap of *C. nodulata, C. petrina*, and *C. necki*. when compared to the *C. pustulosa* complex (Figs 3 and 4). We further tested species boundaries in the *C. nodulata* complex by implementing the coalescent-based species delimitation model STACEY, which aligned with other molecular and morphological assessments, recognizing *C. necki* and *C. petrina* as distinct evolutionary units without *a priori* designation.

Previous researchers questioned the validity of taxa in the *C. pustulosa* complex due to difficulties distinguishing between morphological forms, geographic variants, and distinct species^35–39,46,56,58,62–64^. For example, several species in the *C. pustulosa* complex in western Gulf of Mexico drainages were considered to be sympatric^36,37^. Most recently, the distributions of nominal species in the *C. pustulosa* complex in western Gulf of Mexico drainages have been revised with all species considered allopatric^38^. If each of these “species” are allopatric and restricted to distinct drainages, then phylogenetic analysis should have recoveedr individuals from each drainage as monophyletic. All data and analyses provided a congruent signal and credible evidence that current taxonomy overestimates species-level diversity in the *C. pustulosa* species complex.

Our molecular and morphometric data indicate that current taxonomy overestimates species-level diversity in the *C*. *pustulosa* complex. In fact, our data show greater genetic divergence and morphological distinctiveness between *C. petrina* and *C. necki* than between all *C. aurea*, *C. houstonensis*, *C. mortoni*, *C. pustulosa*, and *C. refulgens* sampled. All five taxa previously recognized as species or subspecies in the *C. pustulosa* species complex exhibited extensive paraphyly (Fig. 4), with no clear distinction between intraspecific variation and interspecific divergence at mtDNA loci (Fig. 5) or clear signals for diagnosis using morphological characters (Fig. 4). With the exception of *C. succissa*, relationships among mtDNA haplotypes show weak associations with currently recognized taxonomy and several nominal taxa share ITS1 haplotypes (Fig. 4). Additionally, morphometric analyses illustrated limited ability to distinguish between members of the *C. pustulosa* complex using shell measurements. Specifically, *C. houstonensis*, *C. mortoni*, *C. pustulosa*, and *C. refulgens* were indistinguishable. The PCA indicated that both *C. aurea* and *C. succissa* were more compressed than other members of the complex (Fig. 4), yet only 74% of individuals identified morphologically as *C. aurea* were binned correctly, with 25% assigned to *C. succissa*. Our molecular-based analyses, however, do not support the recognition of *C. aurea* as a distinct species.

Species within the *C. pustulosa* complex were not molecularly nor morphologically diagnosable, indicating that current taxonomy is vastly overestimating species diversity in this group. Our STACEY analyses were unable to resolve all currently recognized species in the *C. pustulosa* complex as monophyletic entities, which was consistent with our BI and ML analyses. This is likely the result of excessive haplotype sharing and limited sequence divergence at both mtDNA and nDNA loci. Despite MSCs being a powerful approach to account for incomplete lineage sorting in multi-locus data^3,17,65^, our models in STACEY do not support the recognition of more than two species in the *C. pustulosa* species complex.

### Implications for Taxonomy and Conservation

In this study, we used an integrative approach that considered molecular, distribution, and morphology data to evaluate species diversity within *Cyclonaias*. At the species level, congruence across all lines of evidence indicates that current taxonomy overestimates diversity in the *C. pustulosa* species complex. Considering the lack of diagnosis across multiple independent lines of evidence, we consider *C. aurea, C. houstonensis, C. mortoni*, and *C. refulgens* to be synonyms of *C. pustulosa*. This expands the distribution of *C. pustulosa* from the Pascagoula River drainage west to the Nueces River drainage in south Texas (Fig. 6). We do see weak evidence for population structure coinciding to drainage of origin in the *C. pustulosa* complex; however, revising taxonomy such that three or four species are recognized in the *C. pustulosa* complex would result in taxa with extremely disjunct distributions and sympatric species that cannot be distinguished morphologically or genetically. This information could be useful if future management actions are considered for populations of *C. pustulosa*.

Our findings may impact ESA listing decisions by resource management agencies considering that two species (*C. aurea* and *C. houstonensis*) are synonyms of *C. pustulosa*, and another species (*C. petrina* s.s.) contains a cryptic lineage, *C. necki*, which is redescribed herein. Our evaluation of the description of *C. necki* reveals several deficiencies in the work of Burlakova *et al*.^43^. First, their findings were based on only ten specimens from three localities, none of which were collected from the mainstem Colorado or Guadalupe rivers. Thus, their description does not represent the full range of morphological or molecular variation, an important consideration for barcoding^66^. Second, Burlakova *et al*.^43^ report that both *C. necki* and *C. petrina* are closely related to *C. nodulata*; however, no evidence is presented to support this claim and no data or material from *C. nodulata* were examined. In fact, no phylogeny was presented by Burlakova *et al.*^43^, which is necessary to support their claim that *C. necki* has been split from *C. petrina*. Our findings show that the two species (*C. necki* and *C. petrina*) are not sister taxa and reveal that *C. necki* is distinct from both *C. nodulata* and *C. petrina*. The exclusion of a phylogeny fails to capture much about the important evolutionary history of the group and our rigorous phylogenetic approach provides a framework for future evolutionary, ecological, and conservation research. Third, neither the results of the Barcode Index Number designations nor halotype networks were presented despite stating these two methods were used to implement “molecular-based species delimitation,” albeit only from a single mtDNA gene. There has long been concern in the scientific community about taxonomy based on single mtDNA gene^67–69^. Empirical rigor is necessary to ensure confidence in species descriptions and taxonomic stability^18^. Our example illustrates the importance of detailed integrative taxonomy when important management decisions require taxonomic clarity.

## Methods

### Taxon Sampling and Molecular Data

Our taxon sampling concentrated on the following recognized taxa: Cyclonaias asperata (Lea, 1861), *C. aurea, C. houstonensis, Cyclonaias infucata* (Conrad, 1834), *Cyclonaias kleiniana (Lea, 1852), C. mortoni, C. nodulata, C. petrina, C. pustulosa, C. refulgens, and C. succissa*. Efforts were made to sample throughout the range of each species with an emphasis on each type locality. Outgroup taxa were selected based on relationships resolved in previous phylogenetic studies^32,33,70^. All specimens involved with DNA analyses were sacrificed for vouchering in museum collections except four individuals of *C. houstonensis* that were non-lethally swabbed^71^.

Fingings of an undescribed species in the *C. nodulata* complex (voucher UF440979), initially reported by the lead author, were broadly shared with the scientific and regulatory community^51,55^. After submission of our current manuscript for review, the species was named *Cyclonaias necki* by Burlakova *et al*.^43^. We have modified our paper using the name, *C. necki*, and provide a more thorough description based on quantitative morphometrics, robust phylogenetic analyses, and broad taxonomic and geographic sampling.

We utilized two protein-coding mitochondrial (mtDNA) genes and one nuclear (nDNA) locus for phylogenetic reconstruction: *cytochrome c oxidase subunit 1* (CO1), *NADH dehydrogenase subunit 1* (ND1), and *internal transcribed spacer 1* (ITS1). Tissue samples and DNA swabs were preserved in 95% ethanol and DNA was extracted using a modified plate extraction protocol^72^. Primers used for polymerase chain reaction (PCR) and sequencing were as follows: CO1 dgLCO-1490- GGTCAACAAATCATAAAGAYATYGG and CO1 dgHCO-2198-TAAACTTCAGGGTGACCAAARAAYCA^73^; ND1 Leu-uurF- TGGCAGAAAAGTGCATCAGATTAAAGC and LoGlyR-CCTGCTTGGAAGGCAAGTGTACT^32^; ITS1-18S-AAAAAGCTTCCGTAGGTGAACCTGCG and ITS1-5.8S-AGCTTGCTGCGTTCTTCATCG^28^. Thermal cycling profiles for COI were as follows: an initial denaturation at 95 °C for 3 min followed by 5 cycles of 95 °C for 30 s, 45 °C for 40 s, 72 °C for 45 s, then 35 cycles of 95 °C for 30 s, 51 °C for 40 s, 72 °C for 45 s, with a final elongation at 72 °C for 10 min, and hold at 4 °C for 30 min followed by 15 °C forever. Cycling parameters for ND1 and ITS1 follow conditions in original publications^32,73^. The PCR protocol for plate amplifications was conducted in a 12.5 µl mixture: distilled deionized water (4.25 µl), MyTaq^TM^ Red Mix (6.25 µl) (Bioline), primers (0.5 µl) and DNA template (20 ng). Bidirectional sequencing was performed at the Interdisciplinary Center for Biotechnology Research at the University of Florida on an ABI 3730 (Life Technologies). Geneious v 9.1.5^74^ was used to edit chromatograms and assemble consensus sequences. The mtDNA genes were aligned in Mesquite v 3.2.0^75^ using the L-INS-i method in MAFFT v 7.299^76^ and translated into amino acids to ensure absence of stop codons and gaps. The ITS1 alignment was performed using the E-INS-i method in MAFFT to better account for the presence of indels following developers’ recommendations.

### Phylogenetic and Phylogeographic Analyses

We estimated phylogenetic relationships using a concatenated three-locus dataset (i.e. CO1, ND1, ITS1) for members of Quadrulini using maximum likelihood (ML) searches in IQ-TREE v 1.6.1^77,78^ and Bayesian inference (BI) in BEAST v 2.4.8^79^. We used PartitionFinder v2.1.1^80^ to identify partitions and substitution models for IQ-TREE using BIC and to test all models of nucleotide evolution available for BEAST. ML analyses included an initial tree search before implementing 10000 ultrafast bootstrap (UFBoot) replicates to estimate nodal support and nodes are considered supported if they have a UFBoot >95%^81^. BI analyses executed two runs of 3.75 × 10^8^ for a total of 7.5 × 10^8^ generations sampling trees every 10000 generations with an initial 25% burn-in. Trace logs and species trees for the two runs were combined using LogCombiner v 2.4.8. Tracer v 1.6^82^ was used to calculate the standard deviation of log rate on branches and the coefficient of variance was >0.1 for all partitions^45^; therefore, a relaxed log-normal molecular clock was used on all partitions. The relaxed log-normal molecular clock for the first partition was fixed at 1.0 and remaining partitions were estimated by BEAST. Yule process was used as the species tree prior and trees were linked across all partitions. To ensure adequate sampling and proper burn-in, effective sampling (ESS > 200) of all parameters was ensured in Tracer. We used SumTrees v 4.4.0 in DendroPy v 4.4.0^83^ to estimate a consensus tree with an initial 25% burn-in.

TCS haplotype networks^84^ were generated from mtDNA and nDNA independently for each group using PopART 1.7^85^ to visualize the geographic distribution of genetic diversity within and between the members of two species complexes (Fig. 1): the *C. pustulosa species* complex (*C. aurea, C. houstonensis, C. mortoni, C. pustulosa, C. refulgens*, and *C. succissa*) and the *C. nodulata* species complex (*C. necki*, *C. nodulata*, and *C. petrina)*. We included samples lacking ITS1 sequences in the mtDNA haplotype networks to increase sample sizes and expand geographic coverage.

To investigate DNA sequence divergence between and within members of both species complexes, we calculated uncorrected pairwise genetic distances in MEGA7^86^ for CO1, ND1, and ITS1 independently. Uncorrected p-distances were chosen over model-based distances because the later have been shown to inflate OTU assignments^87–89^. Specimens were identified and sequences were grouped according to drainage of collection following recent taxonomic and distributional assessments^38,40,63^: *C. aurea* (Guadalupe and Nueces), *C. necki* (Guadalupe), *C. houstonensis* (Colorado and Brazos), *C. mortoni* (Trinity, Neches, and Sabine), *C. nodulata* (Neches, Red, Sabine, and Mississippi), *C. petrina* (Colorado), *C. pustulosa* (Neosho, Ohio, Osage, Ouachita, Red, St. Croix, and St. Francis), *C. refulgens* (Pascagoula and Pearl), and *C. succissa* (Escambia, Yellow, and Choctawhatchee) (Fig. 6). Gaps and missing data were treated by pairwise deletion between taxa and each taxon was evaluated for diagnostic nucleotides at each mtDNA locus. Additionally, we conducted an analysis of molecular variance (AMOVA)^90^ following 1000 permutations to evaluate inter- and intra-population diversity among members of both the *C. pustulosa* and *C. nodulata* species complexes using ARLEQUIN v 3.5^91^. These groupings align with the null hypothesis based on current taxonomy^35,37,38^ and were not based on distinct genetic groups or phylogeographic results.

### Morphometric Analyses

We collected morphometric data for members of the *C. pustulosa* and *C. nodulata* species complexes by measuring external shell dimensions on all specimens used in genetic analyses and individuals encountered during field surveys. Three morphological measurements were made to the nearest 0.01 mm using digital calipers: maximum length, height, and width. Measurement values were log_e_-transformed to produce a scale-invariant matrix while preserving information about allometry^92,93^. Log_e_-transformed variables were converted into three ratios: height/length, width/length, and width/height. We examined morphological variation using principal components analyses (PCA) in the ggbiplot package^94^ and canonical variates analyses (CVA) in the package Morpho^95^ using R v 3.3.1. The PCA analyses were performed to visualize whether morphological groupings were apparent without *a priori* assignment to a specific group. Canonical variate scores were used for cross-validated discriminant analyses (DA) to measure how reliably morphometric data could assign individuals to each *a priori* defined species in the *C. nodulata* or *C. pustulosa* complex.

### Coalescent-based Species Delimitation

We implemented MSC models using the STACEY v 1.2.2 package^96^ in BEAST v 2.4.8^79^ on two independent molecular matrices coinciding with each species complex. Additionally, we included *Cyclonaias tuberculata* (Rafinesque, 1820) and *C. succissa* as outgroups for the *C. nodulata* and *C. pustulosa* complexes, respectively. The refined matrices were realigned to collapse gaps in ITS1 caused by outgroup taxa. Partitions and substitutions models for each matrix were reevaluated using Partitionfinder v2.1.1^80^. MSC models implemented in STACEY improve efficiency of the DISSECT birth-death collapse model^96^ and infers species boundaries without *a priori* species designations. Therefore, we allowed the model to consider all individuals as separate species and assign individuals to species clusters. Each run executed 3 × 10^8^ generations and logged every 5,000th tree with an initial 10% burn-in. Adequate sampling and proper burn-in was ensured using Tracer. The number of well-supported clusters was calculated using SpeciesDelimitationAnalyser^96^ following an initial 10% burn-in (6,000 trees).

## Electronic supplementary material

Supplementary Figures S1 and S2

Supplementary Table S1

Supplementary Table S2

## Data Availability

All molecular, morphological, and geographic information generated and analyzed as part of this study are publicly available at https://doi.org/10.5066/P9SRSHV2. We included DNA alignment files, morphological measurements, museum catalog numbers, and collection information for every specimen. All DNA sequences analyzed in this study are novel and available on GenBank (MH361762-MH362664, MH633560-MH633655).

## Electronic supplementary material

**Supplementary information** accompanies this paper at 10.1038/s41598-018-33806-z and also publicly available at 10.5066/P9SRSHV2.
