# Supplementary material for: Integrative taxonomy resolves taxonomic uncertainty for freshwater mussels being considered for protection under the U.S. Endangered Species Act
Source: Sci Rep. 2018 Oct 26;8:15892. doi: 10.1038/s41598-018-33806-z (PMC6203750; doi:10.1038/s41598-018-33806-z)
Supplement: Supplementary file 1 — Supplementary Figures S1 and S2 [file 41598_2018_33806_MOESM1_ESM.pdf]

Supplementary Information

Integrative taxonomy resolves taxonomic uncertainty for freshwater mussels being considered for protection under the U.S. Endangered Species Act

Nathan A. Johnson*, Chase H. Smith, John M. Pfeiffer, Charles R. Randklev, James D. Williams, James D. Austin

* Corresponding author at: U.S. Geological Survey, Wetland and Aquatic Research Center, 7920 NW 71st Street, Gainesville, FL 32653, USA. E-mail address: najohnson@usgs.gov

Supplementary Figure S1. The fully resolved maximum likelihood phylogeny with ultrafast bootstrap support values. Taxon labels represent unique identifiers that link metadata (e.g., collection details) and morphological measurements to records in Supplemental Tables S1 and S2, respectively.

Supplementary Figure S2. The fully resolved maximum likelihood phylogeny with posterior probability support values. Taxon labels represent unique identifiers that link metadata (e.g., collection details) and morphological measurements to records in Supplemental Tables S1 and S2, respectively.

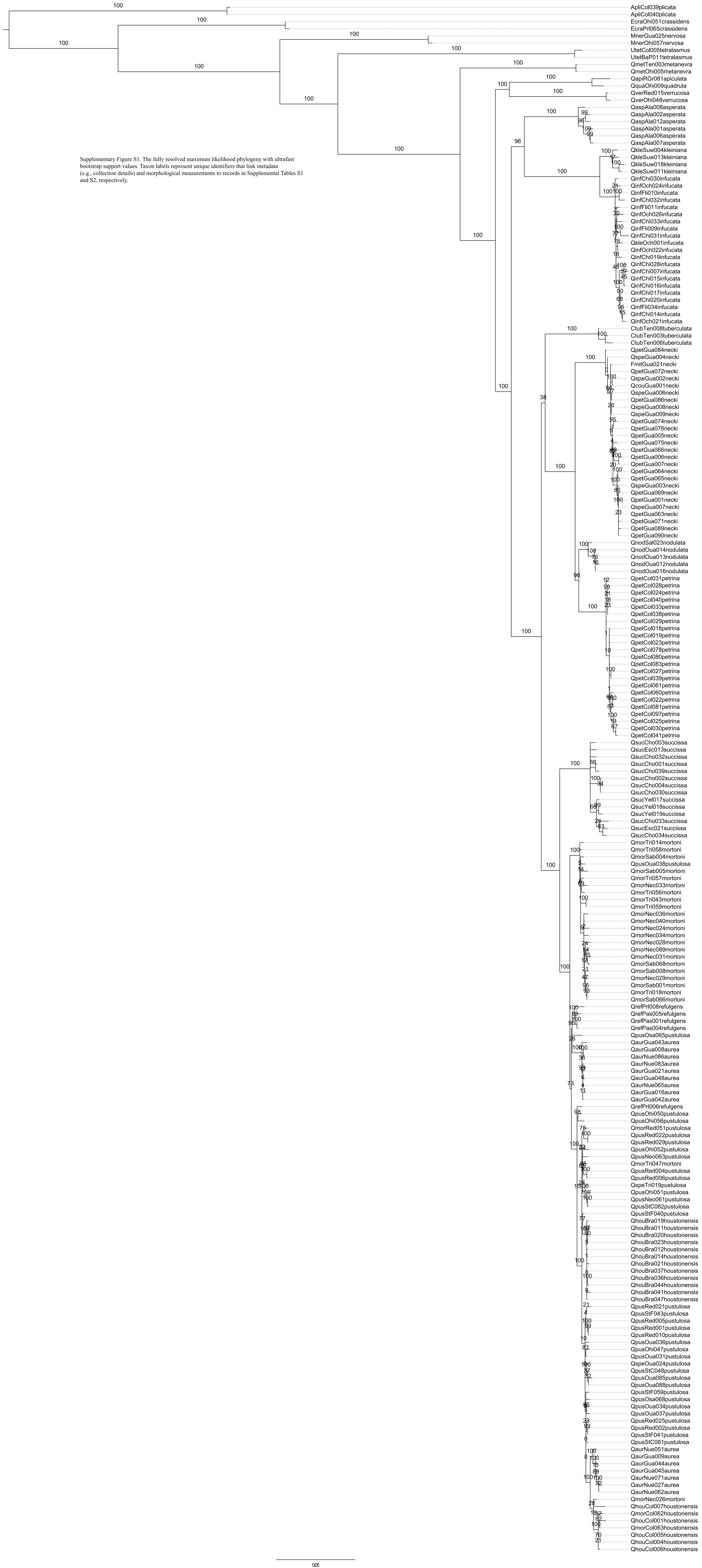

Supplementary Figure S1. The fully resolved maximum likelihood phylogeny with ultrafast bootstrap support values. Taxon labels represent unique identifiers that link metadata (e.g., collection details) and morphological measurements to records in Supplemental Tables S1 and S2, respectively.

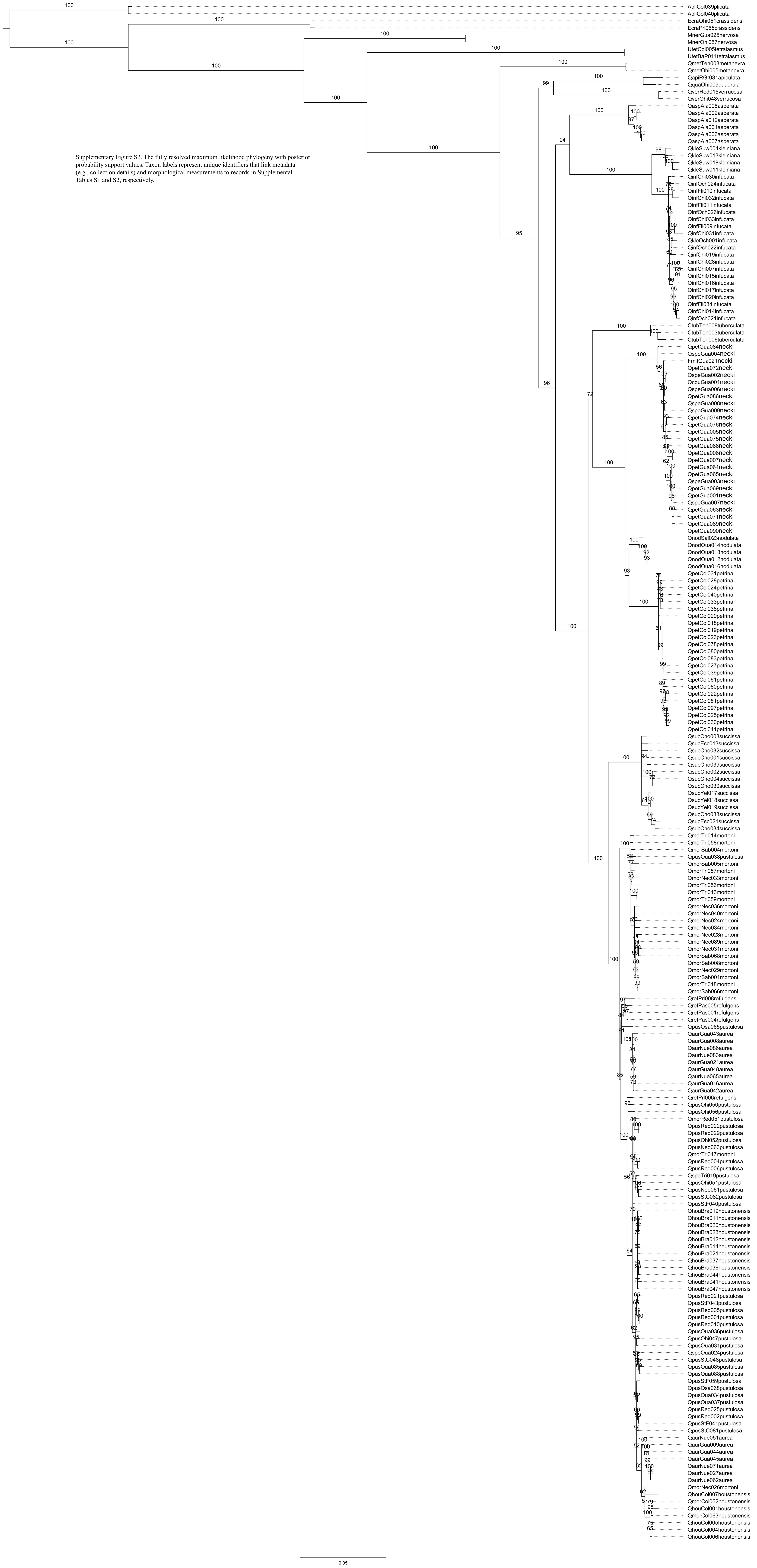

Supplementary Figure S2. The fully resolved maximum likelihood phylogeny with posterior probability support values. Taxon labels represent unique identifiers that link metadata (e.g., collection details) and morphological measurements to records in Supplemental Tables S1 and S2, respectively.
